# Acquisition of threat responses are associated with elevated plasma concentration of endocannabinoids in male humans

**DOI:** 10.1038/s41386-022-01320-6

**Published:** 2022-05-13

**Authors:** Smilla Weisser, Madeleine Mueller, Jonas Rauh, Roland Esser, Johannes Fuss, Beat Lutz, Jan Haaker

**Affiliations:** 1 Department of Systems Neuroscience, University Medical Center Hamburg-Eppendorf, Hamburg, Germany; 2 University Medical Center Hamburg-Eppendorf (Germany), Department of Psychiatry and Psychotherapy, Psychiatry Neuroimaging Branch, Hamburg, Germany; 3 Human Behavior Laboratory, Institute for Sex Research and Forensic Psychiatry, University Medical Center Hamburg-Eppendorf, Hamburg, Germany; 4 Institute of Forensic Psychiatry and Sex Research, Center for Translational Neuro- and Behavioral Sciences, University Duisburg-Essen, 45030 Essen, Germany; 5 Institute of Physiological Chemistry, University Medical Center of the Johannes Gutenberg University, Mainz, Germany; 6 Leibniz Institute for Resilience Research (LIR), Mainz, Germany

**Keywords:** Fear conditioning, Translational research

## Abstract

Endocannabinoids (eCBs) are involved in buffering threat and stress responses. Elevation of circulating eCBs in humans was reported to strengthen inhibition (i.e., extinction) of threat responses and to reduce effects of stressors. However, it remains unclear whether the acquisition of threat responses involves a physiological change in circulating eCBs. Here, we demonstrate in male human volunteers that the plasma concentration of the eCB N-arachidonoylethanolamine (AEA) and its metabolite arachidonic acid (AA) are increased during acquisition of threat responses. Furthermore, elevated responses to a learned threat cue (e.g., rating of fear) were associated with individual increases in plasma concentration of the eCB 2-arachidonoylglycerol (2-AG). In complementing these observations, we found individual increases in AEA associated with elevated neural responses during threat learning in the amygdala. Our results thereby suggest that physiological increases in circulating eCB levels are part of a response mechanism to learned threats.

## Introduction

Endocannabinoids (eCBs) have emerged as a promising target for the pharmacological treatment of anxiety and stress-related disorders. Specific interest in a buffering effect on stress and threat responses by eCB signaling is based on findings derived from experiments in rodents and humans that demonstrated how the enhancement of eCBs dampens behavioral threat responses and their underlying neural processes [1, 2]. eCBs entail N-arachidonoylethanolamine (AEA, anandamide) and 2-arachidonoylglycerol (2-AG), which are both synthesized on demand from arachidonic acid (AA)-containing membrane precursors. AA itself is also a product of eCB degradation. AEA and 2-AG are endogenous ligands to the CB1 and CB2 receptors [3], whereby the presynaptic CB1 receptor is involved in the modulation of behavioral responses by suppression of neurotransmitter release [4].

As such, pharmacological enhancement of eCBs has emerged as an interesting treatment option for individuals suffering from exaggerated threat responses after traumatic experiences. In these individuals, altered concentrations of circulating eCBs have been found [5–7], albeit mixed evidence for enhanced or decreased eCBs, when compared with control cohorts. To understand pathological states and develop pharmacological treatments, information about the physiological response during threat responses of circulating eCBs in humans is needed. In particular, it has not been investigated, whether the concentration of circulating eCBs changes in response to acquisition of threat responses and whether such a change in plasma eCBs is associated with behavioral, physiological, and neurophysiological threat responses.

Laboratory threat responses are commonly examined within fear conditioning models in which humans or other animals undergo acquisition training. This involves the presentation of a neutral stimulus (conditioned stimulus, CS) that is predictive of the occurrence of an aversive, potentially threatening stimulus (unconditioned stimulus, US, e.g., electric shock). As a result, the presentation of the CS elicits a conditioned threat response (CR). Repeated presentation of the CS without the US, so-called extinction training [8], reduces the CR by inhibition of the previously learned CS-US association [9–11]. Acquisition of threat associations has been linked to neural activation in the dorsal anterior cingulate cortex (dACC), the anterior insula (AI), and the amygdala [12–16]. Comprehensive experimental research has confirmed that eCBs are involved in buffering threat responses [4, 17, 18]. In humans, several studies examined the peripheral and central elevation of AEA via a functional polymorphism within the gene coding for a major metabolizing enzyme of AEA, the fatty acid amid hydrolase (FAAH [19–21]). Such an enhancement of circulating AEA plasma concentration was found to dampen the reactivity to negative affect (e.g., threatening images), reduced effects of stressors (e.g., psycho-social stress task) and augmented threat extinction learning [20–24]. These findings align with a recent study in male humans (*N* = 51) exploring the association between circulating AEA levels and neural brain activation during fear extinction. Here, plasma concentration of baseline AEA (start of extinction) was positively correlated to the decrease (exponential decay to the CS+) in neural signaling within brain regions activated during threat acquisition, namely dACC and right AI [25].

While previous evidence supports a role for central and peripheral eCBs in the extinction of threat responses, it has not been investigated in humans whether plasma eCB levels are affected by the acquisition of threats in the first place. Such effect of eCBs on threat acquisition is supported by experiments in rodents, demonstrating a release of AEA in response to aversive electrical foot shocks (compared to low intensity and no shocks) within brain regions that process threats, namely the amygdala, hippocampus, periaqueductal gray and dACC [17, 26]. Thereby, eCB release upon foot shock is assumed to contribute to the conditioned analgesia that is found as a defensive threat response in rodents [27]. Additionally, it was shown that the enhancement of circulating AEA by pharmacological blockade of the FAAH (by URB597) in rodents strengthens acquisition training measured by freezing behavior, when compared to non-shock and saline controls [28]. However, other studies failed to show such an effect of enhanced acquisition training by elevated circulating AEA when comparing genetic polymorphisms of genes coding for the FAAH or pharmacological FAAH inhibition in animals, including humans [20–22, 24].

The function of 2-AG on threat responses seems different from AEA, since increased circulating 2-AG plasma levels by blockade of degrading enzyme monoacylglycerol lipase in rodents has been found to impair fear extinction [29] and to promote fear expression [30]. However, decreases in threat acquisition after elevation of circulating 2-AG plasma levels were also reported in rodents [28]. It is suggested that an optimal 2-AG level is necessary for adaptive threat responses and either too high or too low concentration impairs expression of threat responses [4].

In sum, previous studies that used pharmacological interventions and examined genetic polymorphisms within the eCB system could not reveal consistent effects on the dynamic fluctuations of circulating eCBs during threat acquisition across species. Hence, the key question remains: Do circulating eCB levels change in response to threat acquisition in humans?

To this end, this study examined circulating plasma concentration of the eCBs AEA and 2-AG, as well as AA, before and after a context-dependent threat acquisition that was combined with functional magnetic resonance imaging (fMRI) within a sample of 44 male participants. We hypothesized that circulating eCB and AA concentrations change during the acquisition of fear and that the individual changes in eCB and AA concentration were related to affective ratings of fear, US expectancy, peripheral physiological responses (skin conductance response, SCR), and neural responses (fMRI).

## Methods

### Participants

Fifty healthy male participants gave written informed consent and were reimbursed for participation. Five participants were excluded for the analyzes of eCB and AA plasma concentrations and one additional participant was excluded for fMRI analyses (see Table 1 and Supplementary Methods for details and sensitivity analysis). The study was approved by local ethics committee in Hamburg (Ärztekammer Hamburg).

**Table 1:** Demographics. The final sample consisted of 45 healthy male volunteers (for exclusion criteria of each outcome measure see Supplementary Methods and Results).

Demographics					
Sample (*N* = 45) healthy male volunteers					
Age [years] Mean (SD)	Height [cm] Mean (SD)	Weight [kg] Mean (SD)	Education	Stai-T Mean (SD)	Stai-S Mean (SD)
26.9 (4.2)	181.9 (7.1)	81.4 (11.4)	44,4% university degree 46.7% students without degree	32.7 (7.3)	33.4 (4.9)

### Procedure

**Fig. 1:**
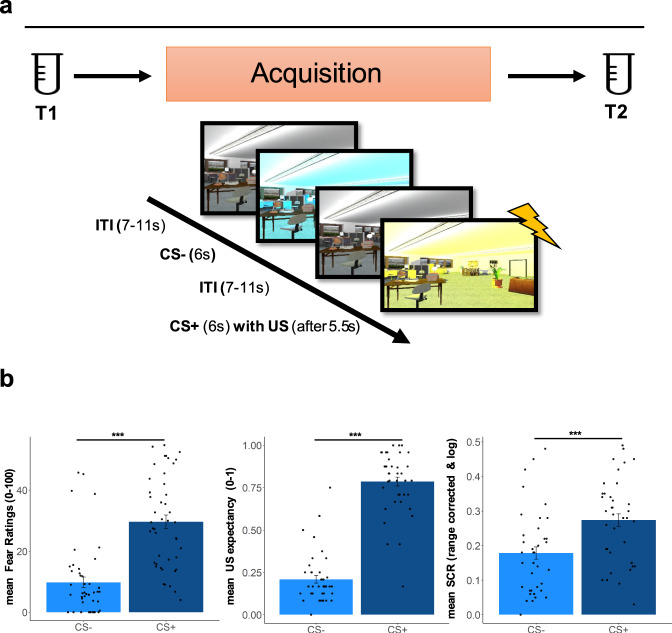
Design and plasma sampling overview. **a** Illustration of the plasma sampling of AEA, 2-AG and AA (T1-T2) during acquisition phase of the context-dependent cue conditioning paradigm. Plasma concentration were sampled before (T1) and directly after (within 5 min) (T2) acquisition training (*N* = 45). Participants also underwent extinction training on Day2 (including eCB sampling) and a retention-test, as well as a reinstatement procedure on Day3, which was part of another study [31] (see Supplementary Methods). **b** Illustration of the mean responses to the CS + and the CS- during acquisition training measured as fear ratings, US expectancy, and skin conductance response (SCR). RmANOVAs of each outcome measure indicated a differentiation between the CS+ and the CS-, with higher responses to the CS + as compared to the CS- (Bonferroni-Holm corrected post-hoc tests all *p*_s_ < 0.001; see Table S1). Analyses of fear ratings and US-expectancy, but not SCRs, further revealed an interaction between CS-type and time (fear ratings (*N* = 45), *p* < 0.001; US-expectancy (*N* = 41), *p* < 0.001; SCR (*N* = 42), *p* = 0.364) reflecting a steeper increase in responses to the CS+, as compared to the CS- during acquisition (see Table S1).

Participants performed a context-dependent cue conditioning paradigm with acquisition training in context A (ACQ, Day1), extinction training in context B (EXT, Day2) and a retrieval-test within a 50:50 mixture of context A and B (generalization context [31], Day3), including a reinstatement procedure. Analyses focused on the ACQ phase, since participants received L-DOPA or placebo (double-blind randomized) before EXT on Day2 (L-DOPA effects, but not eCB analysis is part of a different study [31]). To examine the plasma concentration of AEA, 2-AG, and AA during ACQ, blood samples were taken directly before the ACQ (T1) and directly after the ACQ training (T2), see Fig. 1a. On the second day, blood samples were taken before drug administration (1 h before extinction training, T3), directly before extinction training (T4), and directly after finishing extinction training (T5). No blood sample was taken on the third day (see Fig. S1).

The acquisition training was preceded by a habituation phase (two presentations of each of the CSs within context A and B) without any US. Acquisition training consisted of 24 trials for each CS (duration:6 s), consisting of blue or yellow illuminated rooms (see Supplementary Methods). The CS + was followed by a US in 75% of the trials (5.5 s after CS + onset), consisting of an aversive electrotactile stimulation to the right hand (see Supplementary Methods), whereas the CS- was never followed by a US (see Fig. 1a). Participants were not informed about the conditioning contingencies or the learning element beforehand. Colors of the CS+ and CS- were counterbalanced across participants. Approximately 24 h after conditioning, participants returned to the fMRI laboratory. US electrode was attached, as on day 1 and 24 trials were presented for each CS, while no US was administered. Day 3 was conducted in the psychophysiological laboratory and the US-electrode was attached. The retrieval test consisted of eight unreinforced trials of each CS within a generalization context (50/50-mixture of context A and B). The retrieval test was followed by four unsignaled reinstatement-USs (interval range 10–15 s), while participants were exposed to a black screen. For the reinstatement-USs, the same individual electrical stimulation intensity was used as determined on day 1. 6–10 s after the last reinstatement-US, a second retrieval test (reinstatement-test) was employed, including 16 trials (with no US) of each CS.

### Analyses of AEA, 2-AG, and AA

Blood samples were analyzed for the plasma concentration of N-arachidonoylethanolamine (AEA), 2-arachidonylglycerol (2-AG), and arachidonic acid (AA) as described in [25] and expressed as pmol/mL. Blood samples were collected by repeated venous punctures and immediately centrifuged at 4 °C for 10 min at 2000 *g*. 50 µL of the obtained plasma was aliquoted, frozen immediately, and stored at −80 °C.

### Outcome measurements

#### Fear ratings

Participants rated fear/stress/tension for each CS before and after acquisition training on a computerized Visual Analogue Scale [VAS, 0(none)–100(maximal)], confirmed by key press.

#### US-expectancy

On each CS trial, participants were instructed to rate their US-expectancy as binary choices by pressing the upper (1 = expectancy of a US) or lower key (0 = no expectancy of a US). No scale was presented to the participants to ensure undivided attention (for CS-US contingency awareness see Supplementary Methods).

#### Skin conductance

SCR was measured via self-adhesive Ag/AgCl electrodes, placed on the palmar side of the left hand on the distal and proximal hypothenar. Phasic SCRs to the onsets of each CS were manually scored as the largest response occurring 0.9 to 4.0 s after CS onset. Amplitudes were logarithmized and range-corrected (SCR/SCRmax CS [day]) for separate days to account for inter-individual variability (see Supplementary Methods).

### Statistical analyses

Analyses of the main effects of task were employed by means of repeated measures ANOVAs, using JASP Team (JASP(Version 0.9.1)[Computer software], 2018). In all analyses, an α-level of *p* < 0.05 was adopted and sphericity correction (Greenhouse-Geisser) was applied, if necessary. *P* values were corrected using the Bonferroni-Holm method for independent observations (i.e., plasma concentration of three independent eCBs for each outcome measure). Changes in plasma concentration of AEA, 2-AG, and AA were tested via paired *t* tests between the concentration before (T1) and after (T2) acquisition training, as well as before (T4) and after (T5) extinction training. Association between main effects of task (e.g., CS+- CS- in block2 – block1 on Day1) and changes in eCBs and AA concentration (e.g., difference between T1 and T2) were examined by Pearson correlational analyses (see Supplementary Methods for analysis of extinction and retrieval). In order to control the effects of acquisition for the influence of the circadian rhythm and baseline concentration of eCBs and AA, regression models including these control variables were performed (see Supplementary Methods). Additionally, we examined changes in plasma concentrations within a similar time window (i.e., 60 min) approximately twenty-four hours after acquisition training (for details see Supplementary Methods).

### fMRI acquisition and preprocessing

Task-relevant functional data were obtained on day 1 and day 2 at a 3 T Magnetom-PRISMA System, Siemens, Erlangen, Germany with echo planar multiband imaging with a resolution of 1.5 mm and a 0.5 mm gap. Preprocessing and statistical analysis were employed in SPM12 (Statistical Parametric Mapping, http://www.fil.ion.ucl.ac.uk/spm) including unwarping, realignment, and was coregistered to individual high-resolution structural images. Statistical analyses involved a general linear convolution model at the single-subject level, including onsets for the CS+, CS−, US, introduction, ratings, and button presses. Furthermore, we defined a parametric time modulation of linearly changing responses to the US regressor in order to examine neural responses that decrease as a function of US presentations. Resulting estimate images of interest were then normalized to a sample-customized DARTEL template [32]. Normalized first-level beta-maps were smoothed with an isotropic full-width at half-maximum Gaussian kernel of 4 mm.

Regression models of responses estimates were performed entailing the changes in eCBs and AA concentration and neural responses as the 1) contrast estimates for CS+ > CS- or 2) linearly changing responses to the US. Regions of interest were defined by the main effect during acquisition (without considering the influence of the eCBs and AA), including anatomical masks for the bilateral amygdala and insula [33], as well as the peak voxel in the dACC (MNI (coordinate system of the Montreal Neurological Institute and hospital),*x*;*y*;*z* = 0;28;26) with a surrounding box (20 × 16 × 16 mm). To examine the vmPFC as a key structure for safety learning, defined at the coordinates (MNI:*x*;*y*;*z* = 0;42;−12) with surrounding box (20 × 16 × 16 mm) as in previous experiments [34].

## Results

Participants acquired conditioned responses as evident from fear ratings, trial-wise US expectancy, and SCR during acquisition training (see Table S1; for main effects of extinction training and retrieval see Supplementary Results and Table S11, S13, respectively).

### Increasing AEA and AA plasma concentration during acquisition and extinction training

**Fig. 2:**
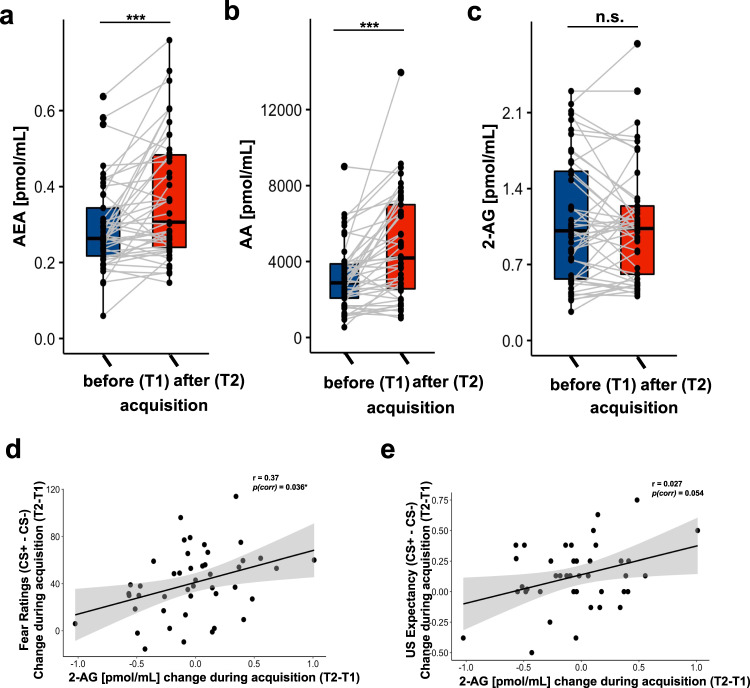
Changes in eCB concentrations during acquisition training. Pair-wise comparisons between **a** AEA, **b** AA, and **c** 2-AG plasma concentration before the acquisition of threat responses (T1, baseline) and after acquisition training (T2). Boxplots illustrate the group concentration average, as well as individual concentration (black point) and their inter-individual change from T1 to T2 (gray lines). Positive correlations reflecting association between the individual increase (from T1 to T2) in differential (CS + -CS-) ratings of fear **d** and expectancy of the US **e** with the increases (from T1 to T2) in plasma 2-AG concentration during acquisition of conditioned threat responses.

Next, we examined the hypothesized changes in eCB and AA plasma concentrations during acquisition training by comparing the within-subject concentrations before the acquisition (T1) with concentrations after acquisition training (T2). Two-sided paired sample t-tests revealed an increase in AEA and AA concentration during acquisition training (*N* = 45; *p*_*s*_ < 0.001, see Fig. 2a, b and Table S3). We found no statistical support for a difference between time points in 2-AG levels (*p* = 0.655, see Fig. 2c and Table S3).

Importantly, the control analyses for the influence of the circadian rhythm revealed no evidence for a change in the plasma concentration of AEA within a similar time window 24 h later (Two-sided paired *t* tests: *t*_*s*_(20) < 1, *p*_*s*_ > 0.3, see Table S4 and Fig. S2a). Hence, it is unlikely that the increase during acquisition training reflects a mere passage of time.

During extinction (*n* = 21, placebo subjects, see *procedure*) we found a general increase in AEA and AA concentration (see Supplementary Results).

### Association between conditioned threat responses and changes in 2-AG plasma concentration during acquisition and extinction training

While our initial analyses revealed an increase in AEA and AA during acquisition of conditioned threat responses, we further examined if individual changes in eCB and AA levels were associated with the individual expression of threat responses. To this end, individual changes in eCB and AA plasma concentration during acquisition training (T2–T1) were tested for association with conditioned threat responses (i.e., block2 – block1 on Day1 of the differential responses to CS+ and CS-). The analyses revealed a positive correlation between 2-AG concentrations and fear ratings, as well as US expectancy, indicating that increasing 2-AG plasma concentration during acquisition training (from T1 to T2) was associated increasing differential fear ratings (CS + -CS-) from beginning to the end of acquisition training (T1 to T2). These findings were mirrored for US-expectancy, albeit lower statistical evidence after correction for multiple comparisons (two-sided Pearson correlation: fear ratings (*N* = 45): *r* = 0.37, *p*_*uncorr*_ = 0.012, *p*_*corr*_ = 0.036; US-expectancy (*N* = 41):*r* = 0.35, *p*_*uncorr*_ = 0.027, *p*_*corr*_ = 0.054, see Fig. 2d, e and Table S5 for separate correlation with each CS). However, we found no support for an association between changes in 2-AG and SCRs (*N* = 42, *p*_*uncorr*_ > 0.5), as well as no correlation between changes in AEA or AA with any of the outcome measurements (all *p*_*uncorr*_ > 0.14, see Table S5). Importantly, none of the eCB or AA changes was associated with the intensitiy or valence of the US (see Table S7). The achieved power for the reported association between fear ratings and changes of 2-AG was moderate 0.72 (see sensitivity analyses in the Supplement).

During extinction, we found a negative correlation, which indicated an association between individual increase in 2-AG concentration and differential decrease in US expectancy from beginning to end of extinction that however not survived correction for multiple comparison (*r* = −0.461, *p*_*uncorr*_ = 0.036, *p*_*corr*_ = 0.108, see Table S12). We found no association between changes in eCB or AA concentration during extinction and responses during the retrieval-test (see Table S14).

### Regression models including control variables support association between conditioned threat responses and changes in plasma concentration

In a second step, we aimed to verify the association between 2-AG and conditioned threat responses within a regression model including baseline concentrations of AEA, 2-AG, and AA, since baseline concentration of AEA has been reported to influence baseline anxiety [35]. To further control for the influence of the circadian rhythm on AEA and 2-AG [36, 37], we included the anticipated change of AEA and 2-AG based on the daytime of T2 as regressors into the model. The regression models for US expectancy and fear ratings revealed again that the individual acquisition of differential conditioned responses (CS + -CS-) was associated with changes in plasma concentration of 2-AG. Hence, a stronger acquisition of conditioned responses was accompanied by a higher increase in 2-AG during acquisition training of fear ratings (*p* = 0.008) and trend-wise for US-expectancy (*p* = 0.056), but not of SCRs. Furthermore, the baseline levels of AEA independently correlated negatively with the acquisition of differential conditioned responses (CS + -CS-) in all outcome measures, indicating that higher baseline AEA levels were associated with lower conditioned responses (all *p*_*s*_ < 0.096). This is in line with a previous study showing a negative association between baseline AEA concentration and anxiety within a (stress) experiment [35]. Importantly, the circadian rhythm of the eCBs (i.e., daytime of the sampling), which was included in each regression model, did not mitigate these effects (see Supplementary Results).

### Regression models on association between neural responses and changes in eCB and AA plasma concentration during acquisition of threat responses

**Fig. 3:**
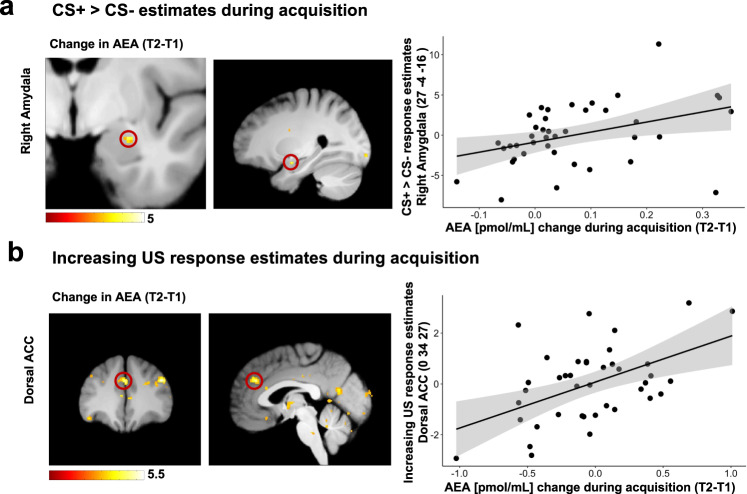
Association between changes in eCB concentrations and neural responses during acquisition training. **a** The regression analysis of neural responses to the CS + , compared to the CS- in the right amygdala revealed a positive association with the changes in AEA plasma concentration during acquisition training (for statistics see Table S9). **b** The regression model of neural responses that increase to the US during acquisition training revealed that an increase in activity in the dorsal ACC was associated with increase in AEA concentration (for statistics see Table S10). T-maps are displayed on an averaged image across the whole sample with a threshold of *p*_*uncorr*_ < 0.005 for illustrative purposes.

Our results already indicated a general increase in AEA and AA during acquisition training, as well as an increase in 2-AG plasma concentration that correlated with differential conditioned responses (fear and US-expectancy ratings). Therefore, we tested via regression models, if the increase in eCB or AA levels is associated with activation in brain regions that reflect the discrimination of learned threat responses (i.e., CS + > CS-) during acquisition training (main effects: Table S8). Analysis revealed a positive association between the differential response (CS + -CS-) in the right amygdala and increasing AEA plasma concentration (T1–T2) during acquisition training (MNI:*x*;*y*;*z* = 27;−4;−16; *t* = 3.89; *p*_*FWE*_ = 0.03; *p*_*uncorr*_ < 0.001, see Fig. 3a and Table S9).

Next, we tested for changes in the eCB system related to neural processes while adapting to the aversive US. Therefore, linear temporal dynamics of neural responses to the US during acquistion training were modeled. Temporal response estimates were then included in a regression model including changes in eCB and AA plasma concentrations (T1 to T2) as regressors (see Table S10). During acquisition training, we found that a linear increase in activation in the left hippocampus was accompanied by increasing plasma concentration of 2-AG (MNI:*x*;*y*;*z* = −15;−9;−20; *t* = 4.7; *p*_*FWE*_ = 0.008; *p*_*uncorr*_ < 0.001). Similarly, we found that linearly increasing activation in the dorsal ACC to the US was associated with increasing plasma concentration of AEA during acquisition training (MNI:*x*;*y*;*z* = 0;34;37; *t* = 4.89; *p*_*FWE*_ = 0.008; *p*_*uncorr*_ < 0.001, see Fig. 3b). Hence, activation in brain regions that are involved in the acquisition of conditioned threat responses, namely the amygdala, hippocampus, and dACC, are associated with increased plasma concentration of 2-AG and AEA.

## Discussion

In this study, we provide support for an overall increase in peripheral concentration of AEA and AA in the blood plasma during the acquisition of conditioned threat responses in male human volunteers. Importantly, no overall increase of 2-AG concentration during acquisition of threat responses was observed, but an association between changes in plasma concentration of 2-AG with the individual expression of conditioned threat responses in fear ratings and a trend in US expectancy. This association of 2-AG change was confirmed in regression models including control factors for the influence of eCB baseline levels [35] and circadian rhythm of eCB concentration [36, 37]. Furthermore, calculated regression analyses of neural responses revealed an association between differential CS responses (i.e., CS + > CS-) in the amygdala and individual increases in AEA concentration during acquisition of threats. In addition, regression analyses revealed an association between individual elevation of AEA and 2-AG concentration and neural responses increasing across US presentations in the hippocampus, as well as the dACC.

Previous experiments in humans and rodents have provided evidence that enhancement of circulating AEA seems to buffer threat responses, in particular during extinction learning [20–24]. In line with these findings are recent results, indicating that plasma levels of AEA before extinction training were associated with decreasing neural responses in brain regions, such as the dACC and the insula, [25]. Our results extend recent findings by showing that the concentration of circulating AEA and its precursor and metabolite AA is already increasing during the acquisition of threat responses, potentially as a normal, physiological function in healthy male humans. Moreover, our results align with previous experiments in rodents, indicating an increase of eCB levels in brain regions such as the amygdala, hippocampus, periaqueductal gray, and mPFC when acquiring threat responses [26, 38]. These studies suggested that the generation of eCBs is a part of a defensive response, which might contribute to conditioned analgesia to foot shocks. Due to the fact that measured blood plasma concentration in humans does not directly reflect the concentration of eCBs in the brain, it is not fully understood from which source circulating eCBs arise and how they specifically reflect eCB driven neural responses [39].

Nevertheless, our results still associate circulating eCBs with processes of aversive learning and related neural responses in the brain. It could well be that the acquisition of differential conditioned threat responses (i.e., higher responses to the CS + as compared to the CS-) probes adaptive response to cope with threats. In fact, individuals suffering from anxiety-related disorders often fail to differentiate between the CS + and the CS- [40, 41]. Hence, the increase in eCBs might be related to the adaptive discrimination between a CS + that predicted the occurrence of the US in comparison to a safe cue as a defensive (coping) response to threats. In parallel, our results might suggest that eCBs are involved in aversive learning in general, since we found elevated plasma concentration during threat acquisition and extinction.

While our results suggest that learning to predict threats is related to increases in eCBs and AA, other factors, such as stress and general arousal (e.g., prior knowledge that aversive stimulation will be applied, positioning in the fMRI environment, etc.) might have additionally contributed to the elevation of eCB concentrations. Prior studies found acute stress related to a decrease in AEA concentration in the rodent brain, whereas mixed results (decreasing, increasing, and no change) in concentration of peripheral AEA were reported in humans [21, 24, 35, 42]. Further studies in rodents reported that acute stress level amplified 2-AG concentration in the amygdala [43], whereas evidence for changes in circulating 2-AG in humans were indecisive (decreasing/no change [35, 39]). However, potential effects of acute stress would rather have affected the general change in eCB concentration and consequentially would neither explain the association between 2-AG and the conditioned threat responses, nor the association between differential (controlled for activation to the CS-) neural activations associated with the individual increase in AEA plasma concentration. Our findings of an association between 2-AG and conditioned threat responses is furthermore in line with a recent study in humans which reported that higher 2-AG concentrations after a traumatic injury predicted greater symptoms of depression 6 months later [44]. This study already suggests that changes in the physiological concentration of eCBs are relevant to the adaption of future behavior.

Seemingly in contrast to our results, other studies in humans did not show an effect of enhanced eCB levels on the performance during acquisition training using polymorphisms of genes coding for the FAAH or pharmacological inhibition of the FAAH [20, 24, 45]. However, an absence of enhanced behavioral or physiological measures in acquisition by pharmacologically augmented eCB level does not necessarily contradict that physiological responses of the eCB system are involved during acquisition of threats. We advocate for a better understanding how the eCB system is involved during the acquisition of threat responses. These insights might aid to understand disturbances of the eCB system in individuals that experienced traumatic events [6, 7, 42] and provide a basis to develop new treatments for trauma and stress-associated disorders.

Our findings are limited by the investigation of male volunteers only. Future studies are warranted to delineate the eCB responses to threats in female populations, given that females are overrepresented in populations that suffer from anxiety-related disorders [46].

Our results indicate that acquisition of threat responses is reflected in dynamic changes of eCB plasma concentrations, by elevated plasma concentration of AEA and its metabolite AA during acquisition of threats. We further provide initial evidence for an association between increased 2-AG plasma concentration with fear ratings as well as between increases in AEA concentration and elevated activity in the amygdala. Hence, our results provide a novel perspective of how physiological changes in circulating eCBs are involved in aversive learning. We further suggest future studies to reveal the potential of eCBs in adaptive and maladaptive coping with threats and thereby advancing pharmacological treatment that focuses on balancing eCB plasma in patients with anxiety-related disorders.

## Supplementary information

Acquisition of threat responses is associated with elevated plasma concentration of endocannabinoids in male humans

## Data Availability

Source data for the analyses and figures are available at: https://osf.io/vq3bs/?view_only=ea7da978554f43bca27b89059e04d7e6
