# Supplementary material for: Acquisition of threat responses are associated with elevated plasma concentration of endocannabinoids in male humans
Source: Neuropsychopharmacology. 2022 May 13;47(11):1931–8. doi: 10.1038/s41386-022-01320-6 (PMC9485143; doi:10.1038/s41386-022-01320-6)
Supplement: Supplementary file 1 — Acquisition of threat responses is associated with elevated plasma concentration of endocannabinoids in male humans [file 41386_2022_1320_MOESM1_ESM.pdf]

Supplementary Materials

Acquisition of threat responses are associated with elevated plasma concentration of endocannabinoids in male humans

Supplementary Methods

Participants

Fifty healthy, right-handed male adults without any self-reporteded life-time psychiatric or neurological diagnoses (age: range: 20 – 38 years, *M*=26.9, *SD*=4.2), were recruited in this study. One subject had to be excluded after illegal drug-screening test (positive drug urine test), carried out prior to acquisition training on day1 (M-10/3-DT; Diagnostik Nord). Four additional subjects were excluded for the statistical analyses (incidental finding of a brain abnormality N=1, not following the instructions N=1, accidental press of the emergency bell N=1, missing blood samples N=1). The final sample for the analyses of blood and behavioral data included forty-five participants. One additional subject had to be excluded for fMRI analyses, because of movement-related artefacts in the scanner. The final sample included in fMRI analyses therefore contained fourty-four subjects. Participants gave written informed consent and were reimbursed with 120 EUR.

The participants were recruited to participate at a pharmacological intervention during extinction [1], as well as examination of eCB and AA concentration during acquisition and extinction training.

Sensitivity analyses

Post-hoc calculated sensitivity analyses of a two-sided, paired comparison for plasma concentration indicated a sufficial sample size of 45 participants to detect an effect size dz = 0,4941242 and a critical t =2,0153676 assuming a power (1-β error probability) of 0.90 and an α error probability of 0.05 (G*Power 3.1). Similar analyses for correlation analyses indicated that the sample of 45 participants would be sufficient to detect correlation with a ρ=0,46 and a critical r=0,29 assuming a power (1-β error probability) of 0.90 and an α error probability of 0.05 (G*Power 3.1). Post-hoc sensitivity analyses for a two-sided correlation analyses for a sample size of 21 participants, as available in the extinction training, revealed a rather reduced power (resulting power =0.379, assuming an alpha rate of 0.05 and an spearman-rho of 0.355, computed with G*Power 3.1).

Stimulus material

Conditioned stimuli. Computer-generated pictures showing office-rooms (Source Engine, Valve Corporation, Bellevue, USA) were used as context stimuli (presented as intertrial-intervals ITIs, duration range:7-11sec, mean:7.8sec), illuminated in either a blue or a

yellow light (duration:6sec) thus serving as CSs. The visual stimulus material was presented on a computer screen using Presentation® software (NeuroBehavioral Systems, Albany California, USA).

Unconditioned stimulus. An electrotactile stimulation administered to the right dorsal hand (3 pulses of 2ms duration with a 50ms interval) served as the US and was presented 5.5sec after CS+ onset (delivered by a DS7A electrical stimulator, Digitimer, Welwyn Garden.

Outcome measurements

Fear ratings. No participants were excluded from the analyses [excluded participants: N(day1)=0, resulting sample size N(day1)=45].

US-expectancy. Participants were excluded from the analyses (day-wise) if more than one third of all data points were missing [excluded participants: N(day1)=4, resulting sample size N(day1)=41]. At the end of the experiment, CS-US contingency awareness was assessed using a semi-structured interview [2] and based on these results 37 participants were classified as aware and 5 were classified as unaware of CS contingency.

Skin conductance. Data were recorded with a BIOPAC MP-100 amplifier (BIOPAC Systems Inc, Goleta, California, USA) using AcqKnowledge 4 software. Then, data were down sampled using a custom-made computer program (EDAview, Version 1.0) to 10 Hz. Phasic skin conductance responses (SCRs) to the onsets of each CS were manually scored defined as the largest response occurring within a time window between 0.9 to 4.0 s after CS onset. Non reactions were scored as zeros and trials with obvious electrode artefacts were scored as missing data. SCR data from a limited number of participants revealed insufficient data quality (as judged by two researchers; due to signal-disturbances by the fMRI acquisition) and were consequentially excluded (day-wise) prior to data analyses [excluded participants: N(day1)=3, resulting sample size N(day1)=42].

Statistical analyses

Main effects of task. Analyses of the main effects regarding fear ratings, US-expectancy and SCR (descriptives see Table S2; Correlation of US-intensity and US-valence see Table S7) were employed by repeated measures ANOVAs (rmANOVAs). RmANOVAs included a within-subject factor for the CS-type (CS+ and CS-) and the effect of time (ACQ: fear ratings: 2 levels that include ratings before and after acquisition training, SCR and US-expectancy: 3 levels for each block that represent an average across 8 trials; EXT: fear ratings: 2 levels that include ratings before and after extinction training, SCR and US-expectancy: 3 levels for each block that represent an average across 8 trials; retrieval-test: fear ratings: 2 levels that include ratings after extinction training and before retrieval-test, SCR and US-expectancy: 2 levels for the last block of EXT and the first block of the retrieval-test, that represent an average across 8 trials).

Association between main effects of task and changes in AEA, 2-AG and AA *concentration.* Analyses applied focus on changes in AEA, 2-AG and AA during acquisition training, extinction training and retrieveal-test and a potential association with the main effect of task for each outcome measure. For the acquisition phase indices reflecting the main effect of tasks during acquisition (fear ratings: CS+ - CS- in block2 – block1, US-expectancy and SCR: CS+ - CS- in block3 – block1) were tested for a correlation with changes in AEA, 2-AG and AA (difference between T1 and T2). For the extinction training indices reflecting the main effect of tasks during extinction (fear ratings: CS+ - CS- in block2 – block1, US-expectancy and SCR: CS+ - CS- in block3 – block1) were tested for a correlation with changes in AEA, 2-AG and AA (difference between T5 and T4). For the retrieval-test indices reflecting the main effect of tasks of the retrieval-test (fear ratings: CS+ - CS- in block1 on day3 – block2 on day2, US-expectancy and SCR: CS+ - CS- in block1 on day3 – block3 on day2) were tested for a correlation with changes of AEA, 2-AG and AA during extinction (difference between T5 and T4). Again, an α-level of $p<0.05$ was applied and p-values were corrected using the Bonferroni-Holm method for three independent observations (i.e., plasma concentration of three independent eCBs for each outcome measure). Additional data for the analyses of the association between the AEA, 2-AG and AA and the CS+ and CS- responses (instead of the CSs difference), separately calculated for each outcome measure can be found in Table S5 for ACQ, Table S12 for EXT and Table S14 for retrieval-test.

Regression models of behavioral responses during acquisition of threat responses and changes in AEA, 2-AG and AA plasma concentration. Indices reflecting the main effect of task (e.g., CS+ - CS- in block2 – block1), were entered into linear regression models (separately calculated for each outcome measure) including changes in for AEA, 2-AG and AA as a separate regressors. Linear regression models used backward selection of regressors with an α-level of $p<0.1$. Furthermore, regressors for the baseline concentration of AEA, 2-AG and AA (T1) reflecting the expected impact of circadian rhythmic on the concentration of AEA and 2-AG were included in regression models.

Daytime of blood sampling. Previous studies in humans have shown that eCB plasma concentration follows a circadian rhythm and hence, changes during the day-time. Hanlon et al. (2020) reported a differential circadian rhythmic for 2-AG and AEA documenting the relative level of both in percentage respective to the 24-hour mean over the day [3]. The time of blood sampling in our study was coded for 2-AG (values from 1.00 to 1.60 in steps of 0.05) and AEA (values from 0.80 to 1.30 in steps of 0.05), by interpolation steps of 30 min that would reflecting the anticipated, relative changes in individual concentrations from the individual 24-hr mean, using previously reported results [3,4]. To control for the circadian rhythm, these anticipated

influences of daytime for the sample T2 was entered as two regressors (one for AEA, one for 2-AG) into each regression model.

Baseline level of AEA, 2-AG and AA. Studies have shown that inter-individual differences in baseline levels of eCBs exist and that these baseline level might correlate with anxiety during the experiment [5]. To examine the effect of baseline levels of AEA, 2-AG and AA (i.e., T1 on day 1) on dependent measures, baseline levels were entered as regressors in each regression model.

fMRI analyses. MRI data were obtained on day 1 and 2 at a 3T Magnetom-PRISMA System (Siemens, Erlangen, Germany) using a 64-channel head coil and parallel single-shot echo-planar imaging (GRAPPA, in-plane acceleration factor 2) [6]and simultaneous multi-slice acquisitions ("multiband", slice acceleration factor 2 [7,8] as described in [9]). Image reconstruction algorithm was provided by the University of Minnesota Center for Magnetic Resonance Research. Echo planar multiband images were acquired with 42 continuous axial slices (1.5 mm thickness, 0.5 mm gap) using a T2*-sensitive sequence (TR = 1493 ms, TE = 30 ms, flip angle = 60°, field of view = 225 × 225 mm^2). Slice arrangement was individually adjusted in order to cover the following areas: dorsal anterior cingulate cortex, ventral medial prefrontal cortex, nucleus accumbens, amygdala, and midbrain SN/VTA. Moreover, high-resolution T1-weighted structural brain image (MP-RAGE sequence, 1 mm isotropic voxel size, 240 slices) were obtained. To account for T1 equilibrium effects, the first five functional images of the time series collected during acquisition training (day 1) were discarded.

To examine associations between neural responses in regions that were responding to the main effects of task with the changes in AEA, 2-AG and AA, individual contrast estimate maps for higher responses to the CS+ as compared to the CS- were included into group analysis using one sided t-test models, as implemented in SPM. We employed four separate regression models that all included individual changes in AEA, 2-AG and AA concentration as well as an intercept as regressors. Hence, we are able to predict neural responses that were either 1) the contrast estimates for CS+>CS- or 2) linearly changing responses to the US presentations.

Supplementary Results

Acquisition training

Regression models including control variables support association between conditioned threat responses and changes in plasma concentration during acquisition.

Fear Ratings. In line with single correlation analysis, regression analysis revealed a significant model for the change in differential fear ratings across acquisition (CS+ - CS- in

block2 – block1, N=45; $F(3, 41)=4.51$, $p=0.008$, adjusted $R^2=0.193$) included a positive association with the change of 2-AG during acquisition (T2-T1; t=2.84, $p=0.007$) and a negative association with the baseline levels of AEA (t=-2.41, $p=0.021$). This indicates that individuals with higher baseline plasma concentration of AEA reported lower differential fear during the acquisition training. A similar association has been reported in an experiment of psychosocial stress in humans [5]. Our regression model furthermore included a positive association between the baseline level of AA (t=2.16, $p=0.036$), which indicates that individuals with higher baseline plasma concentration of AA reported higher differential fear during acquisition training.

US-expectancy. Similar to the fear ratings, a significant regression model (CS+ - CS- in block3 – block1, N=41; $F(3, 37)=3.71$, $p=0.015$, adjusted $R^2=0.231$) for the differential US-expectancy across acquisition (T2-T1) included a positive association with the change of 2-AG during acquisition (T2-T1; t=1.98, $p=0.056$). In line with fear ratings, the model for US-expectancy further suggests support for a negative association with the AEA baseline level (t=-1.71, $p=0.096$). Even though both results just barely missed significance, results are similar to findings in fear ratings. The regression model further provided support for a negative association of the anticipated circadian changes in 2-AG concentration (t=- 2.253, $p=0.030$).

SCR. We found a trend towards a regression model for change in differential SCRs during acquisition training (CS+ - CS- in block3 – block1, $F(1, 40)=6.87$, p=0.012, adjusted $R^2=0.159$). In line with the correlational analyses, we found no statistical support for an association between SCRs and the changes in 2-AG. However, a negative association between SCRs and baseline concentration of AEA (t=-2.62, $p=0.012$) was found. Hence a lower behavioral fear response is associated with increased baseline concentration levels of AEA, which mirrors the regression models for fear ratings and US-expectancy.

Association between trait and state anxiety and changes in AEA, 2-AG and AA plasma concentration.

Post-hoc calculated explorative analyses testing for a potential association between individual anxiety level (State and Trait Anxiety Inventory, STAI) and eCB related blood plasma concentration changes according to acquisition training revealed a positive correlation between trait anxiety and the changes of AA plasma concentration ($r=0.33$, $p_{uncorr}=0.025$, see Table S6) during the acquisition training (T2-T1) and, albeit lower statistical support, also for the changes of AEA plasma concentration ($r=0.29$, $p_{uncorr}=0.055$, see Table S6) during acquisition training. The results might indicate that individuals with higher trait anxiety scores showed a stronger increase in AEA and AA plasma concentration during acquisition. Importantly, we did not correct these analyses for multiple comparisons, since these analyses were exploratory. We found no support of an association between baseline levels of AEA, 2-

AG or AA with trait or state anxiety (p_{uncorr}>0.05, see Table S6). Taking together, there is initial support for an association between trait anxiety and the changes in AA concentration (and to some extend for AEA) during acquisition (T2-T1).

Extinction training

Main effects of task during extinction training.

Post-hoc calculated exploratory analyses of changes in AEA, 2-AG and AA during extinction learning included a reduced number of participants on day 2 (plasma concentration: N=21, fear ratings: n=21, US-expectancy: n=21, SCR: n=17, i.e. only participants in the placebo group; no effect of placebo pill on eCB plasma levels, see figure S2 and table S4). Over the time course of extinction training, participants still exhibited conditioned responses, measured as fear ratings, US-expectancy and SCR, indicated by a main effect of CS-type in the rmANOVAs (main effect of CS-type: fear ratings(N=21): F(1, 20)=15.66, *p*<0.001, η^2=0.27; US-expectancy(N=21), F(1,20)=11.42, *p*=0.003, η^2=0.18; SCR(N=17): F(1,16)=4.49, *p*=0.050, η^2=0.22, see table S11 for full statistics), with higher responses to the CS+ as compared to the CS- (Bonferroni-Holm adjusted post-hoc tests for CS+ - CS-: fear ratings: t(40)=3.96, p<0.001; US-expectancy: t(40)=3.38, *p*=0.003, SCR: t(32)=3.24, *p*=0.005). Analyses of all outcome measures further revealed an interaction between CS-type and time (fear ratings(N=21): F(1,20)=22.64, *p*<0.001, η^2=0.07; US-expectancy(N=21): F(1.81,20)=13.69, *p*<0.001, η^2=0.06; SCR(N=17): F(0.49,0.38)=20.44, *p*<0.001, η^2=0.56), which reflected a steeper decrease in responses to the CS+ during extinction (Bonferroni-Holm adjusted post-hoc tests: fear ratings: $t_{CS+,Block1\ -\ CS+,Block2}$(78)=5.42, *p*<0.001; $t_{CS-,Block1\ -\ CS-,Block2}$(78)=0.58, *p*=1; US-expectancy: $t_{CS+,Block1\ -\ CS+,Block3}$(122)=7.49, *p*<0.001; $t_{CS-,Block1\ -\ CS-,Block3}$(122)=1.94, *p*=0.839; SCR: $t_{CS+,Block1\ -\ CS-,Block3}$(98)=8.42, *p*<0.001; $t_{CS-,Block1\ -\ CS-,Block3}$(98)=6.05, *p*<0.001) (see Table S11 for full statistics).

Main effects of eCB and AA change during extinction training.

RmANOVAs were conducted (placebo group only, N=21) to test for an effect of blood plasma concentration changes of AEA, 2-AG and AA during the time course of extinction training (before extinction, before intake of the placebo pill: T3, before extinction, after the intake of the placebo pill: T4, after extinction: T5). The analyses revealed a main effect of time for AEA (F(1.44,28.88)=4.42, *p*=0.018, *f*=0.47) and AA (F(1.54,30.89)=22.96, *p*<0.001, *f*=1.07). Bonferroni-Holm adjusted post hoc tests further showed an increase (*p*<0.01) for plasma concentration of AEA and AA when comparing concentration before and after extinction training (from T4 to T5; AEA (T4: *M*=0.320 , *SD*=0.148, T5: *M*=0.379 , *SD*=0.141) and AA (T4: *M*=3677 , *SD*=2248, T5: *M*=6410 , *SD*=3732). Furthermore an increase (*p*<0.01)

in AEA and AA plasma concentration was found, when comparing baseline levels on day2 (T3, before placebo administration) with plasma concentration after extinction training (from T3 to T5, AEA (T3: M=0.296 , SD=0.122, T5: M=0.379 , SD=0.141) and AA (T3: M=2167 , SD=2248, T5: M=6410 , SD=3732). There was no evidence for an increase (p>0.05) from baseline on day 2 to the time-point before extinction training (Mean difference (T4-T3): AEA=0.003, AA=-410.3). Consistent with results for acquisition training, we found no support for changes in 2-AG plasma concentration ($F(1.22,24.48)=0.34$, $p=0.612$, $f=0.13$). In summary, we found an increase in AEA and AA plasma concentration during extinction training. The correlation analysis of eCBs and AA with extinction effect, revealed no significant association that survived correction for multiple comparison (see Table S12).

In summary, we found an increase in AEA and AA plasma concentration during extinction training.

Retrieval-Test

Main effects of task during retrieval-test.

Post-hoc calculated exploratory analyses of the main effect of tasks of the retrieval-test and changes in AEA, 2-AG and AA during extinction (T5-T4) included a reduced number of participants on day 3 (plasma concentration: N=21, fear ratings: n=21, US-expectancy: n=21, SCR: n=17, i.e. only participants in the placebo group). Over the time course of the retrieval-test, participants exhibited conditioned responses, measured as fear ratings, US-expectancy and SCR, indicated by a main effect of CS-type in the rmANOVAs (main effect of CS-type: fear ratings(N=21): $F(1, 20)=14.79$, $p=0.001$, $\eta^2=0.28$; US-expectancy(N=21), $F(1,20)=11.04$, $p=0.003$, $\eta^2=0.18$; SCR(N=17): $F(1,16)=11.38$, $p=0.004$, $\eta^2=0.1$, see table S13 for full statistics), with higher responses to the CS+ as compared to the CS- (Bonferroni-Holm adjusted post-hoc tests for CS+ - CS-: fear ratings: $t(40)=3.85$, $p<0.001$; US-expectancy: $t(40)=3.32$, $p=0.003$, SCR: $t(32)=3.37$, $p=0.004$). Analyses of all outcome measures further revealed an interaction between CS-type and time (fear ratings(N=21): $F(1,20)=6.95$, $p=0.016$, $\eta^2=0.03$; US-expectancy(N=21): $F(1,20)=6.19$, $p=0.022$, $\eta^2=0.04$; SCR(N=17): $F(1, 16) = 5.12$, $p=0.038$, $\eta^2=0.02$), which reflected an increase in responses to the CS+ in the retrieval-test (Bonferroni-Holm adjusted post-hoc tests: fear ratings: $t_{CS+,EXT – CS+,retrieval}(78)=-2.73$, $p=0.059$; $t_{CS-,EXT – CS-,retrieval}(78)=0.30$, $p=1$; US-expectancy: $t_{CS+,EXT – CS+,retrieval}(122)=-3.55$, $p=0.006$; $t_{CS-,EXT – CS-,retrieval}(122)=-0.44$, $p=1$; SCR: $t_{CS+,EXT – CS-,retrieval}(98)=-3.42$, $p=0.016$; $t_{CS-,EXT – CS-,retrieval}(98)=-1.80$, $p=0.517$).

Association between conditioned threat responses and changes in eCB and AA change during retrieval-test.

The correlation analysis of the main effects of the retrieval-test and changes of eCBs and AA during extinction (T5-T4), revealed no significant association that survived correction for multiple comparison (see Table S14).

References

[1] Esser R, Korn CW, Ganzer F, Haaker J. L-DOPA modulates activity in the vmPFC, nucleus accumbens, and VTA during threat extinction learning in humans. eLife 2021;10.

[2] Bechara A, Tranel D, Damasio H, Adolphs R, Rockland C, Damasio AR. Double Dissociation of Conditioning and Declarative Knowledge Relative to the Amygdala and Hippocampus in Humans. Science 1995;269(5227):1115–8.

[3] Hanlon EC. Impact of circadian rhythmicity and sleep restriction on circulating endocannabinoid (eCB) N-arachidonoylethanolamine (anandamide). Psychoneuroendocrinology 2020;111:104471.

[4] Hanlon EC, Tasali E, Leproult R, Stuhr KL, Doncheck E, Wit H de et al. Circadian Rhythm of Circulating Levels of the Endocannabinoid 2-Arachidonoylglycerol. The Journal of Clinical Endocrinology & Metabolism 2015;100(1):220–6.

[5] Dlugos A, Childs E, Stuhr KL, Hillard CJ, Wit H de. Acute Stress Increases Circulating Anandamide and Other N-Acylethanolamines in Healthy Humans. Neuropsychopharmacol. 2012;37(11):2416–27.

[6] Griswold MA, Jakob PM, Heidemann RM, Nittka M, Jellus V, Wang J et al. Generalized autocalibrating partially parallel acquisitions (GRAPPA). Magn. Reson. Med. 2002;47(6):1202–10.

[7] Moeller S, Yacoub E, Olman CA, Auerbach E, Strupp J, Harel N et al. Multiband multislice GE-EPI at 7 tesla, with 16-fold acceleration using partial parallel imaging with application to high spatial and temporal whole-brain fMRI. Magn. Reson. Med. 2010;63(5):1144–53.

[8] Feinberg DA, Moeller S, Smith SM, Auerbach E, Ramanna S, Glasser MF et al. Multiplexed Echo Planar Imaging for Sub-Second Whole Brain FMRI and Fast Diffusion Imaging. PLoS ONE 2010;5(12):e15710.

[9] Setsompop K, Gagoski BA, Polimeni JR, Witzel T, van Wedeen J, Wald LL. Blipped-controlled aliasing in parallel imaging for simultaneous multislice echo planar imaging with reduced g -factor penalty. Magn. Reson. Med. 2012;67(5):1210–24.

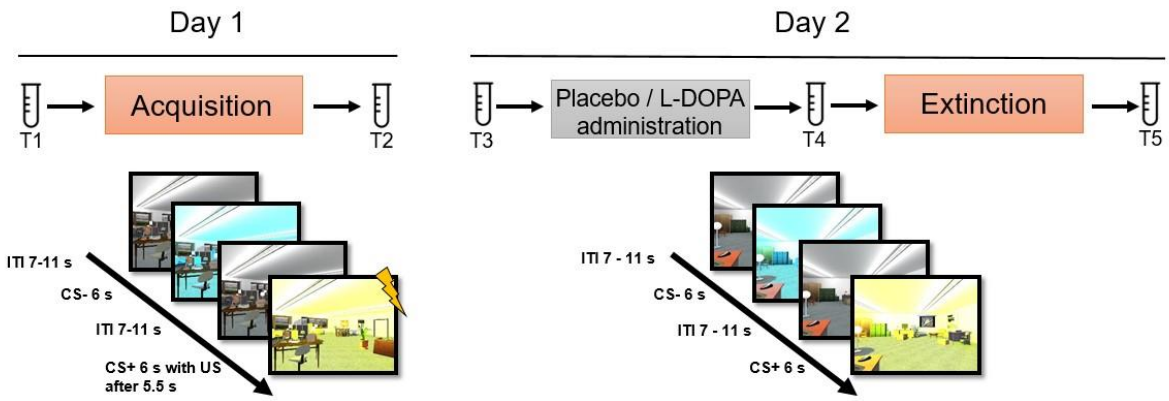

Figure S1. Experimental design: Timeline of blood plasma sampling of AEA, 2-AG and AA (T1-T5). Plasma concentrations were sampled before (T1) and after (T2) acquisition training (N=45) on the first day. Participants underwent extinction training on Day 2 (including eCB sampling T3-T5; part of another study). The acquisition and extinction training consisted each of 24 trials per CS (reinforcement rate of CS+: acquisition training 75%, extinction training 0%). Presentation times for ITIs and CSs are depicted next to the pictures.

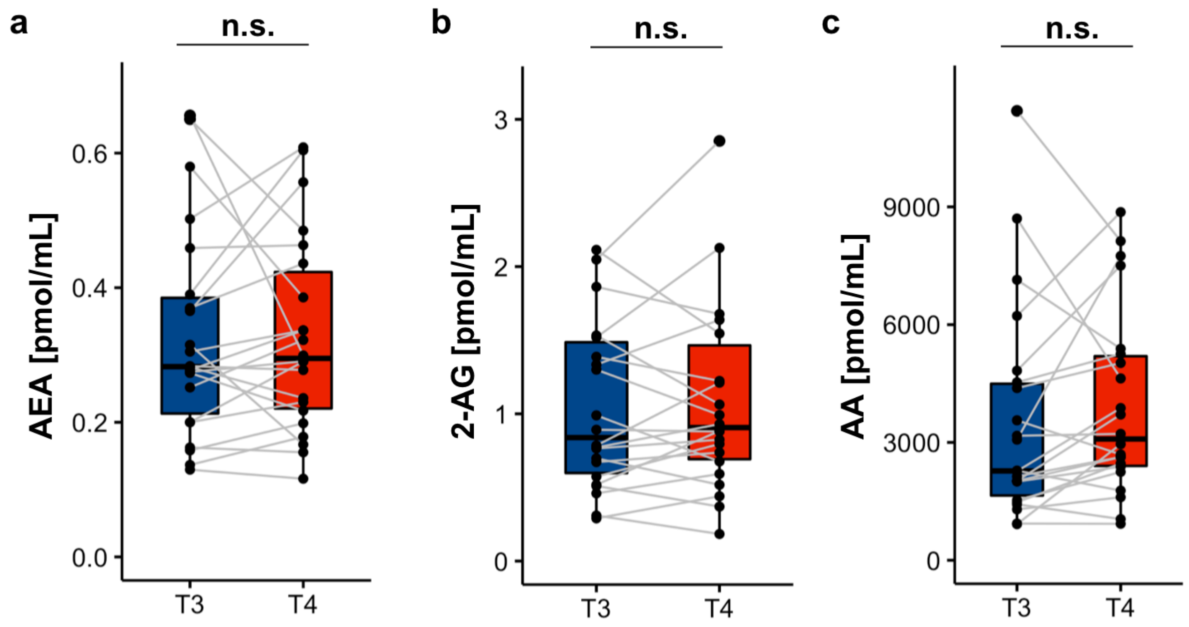

Figure S2. Illustration of pair-wise comparisons examining plasma concentration of **a)** AEA, **b)** 2-AG and **c)** AA before ingestion of a placebo pill (T3) and after (i.e., 60min) ingestion of a placebo pill (T4) twenty-four hours after acquisition training. n.s.=not significant, i.e. $p>0.05$

Table S1. Main effect of task during acquisition. Repeated-measures ANOVA showing conditioned responses of fear ratings, trial-wise US-expectancy ratings and skin conductance responses (SCR) during acquisition training.

Main effects of task during acquisition					
**effect**	**measure**	**N**	**F(df)**	***p***	**η^2**
CS-Type	Fear Ratings	45	(1, 44) = 74.47	<0.001***	0.63
	US-expectancy	41	(1, 40) = 184.83	<0.001***	0.82
	SCR	42	(1, 41) = 47.41	<0.001***	0.17
Time	Fear Ratings	45	(1, 44) = 77.24	<0.001***	0.64
	US-expectancy	41	(1.87, 74.96) = 0.66	.511	0.02
	SCR	42	(1.42, 58.09) = 6.16	0.009**	0.07
Time * CS	Fear Ratings	45	(1, 44) = 93.81	<0.001***	0.68
	US-expectancy	41	(1.83, 73.22) = 12.18	<0.001***	0.23
	SCR	42	(1.96, 80.27) = 1.02	0.364	0.01

*Note. The label * marks results if p < .05, the label ** marks results if p < .01, the label *** marks results if p < .001*

Table S2. Descriptives Day 1. Means and standard errors of fear ratings, trial-wise US-expectancy ratings and skin conductance responses during acquisition training (fear ratings include ratings before and after acquisition training; SCR and US-expectancy: each block represents an average across 8 trials).

**Descriptives Day 1**				
**measure**	**Time**	**CS**	***M***	***SE***
Fear Ratings	B1	CS+	9.78	11.95
		CS-	10.09	12.55
	B2	CS+	49.59	24.70
		CS-	9.50	15.34
	Mean Day 1	CS+	29.68	14.94
		CS-	9.79	11.83
US-expectancy	B1	CS+	0.75	0.24
		CS-	0.27	0.19
	B2	CS+	0.83	0.18
		CS-	0.17	0.16
	B3	CS+	0.79	0.21
		CS-	0.19	0.16
	Mean Day1	CS+	0.79	0.18
		CS-	0.21	0.15
SCR	B1	CS+	0.29	0.13
		CS-	0.22	0.14
	B2	CS+	0.26	0.15
		CS-	0.15	0.13
	B3	CS+	0.23	0.15
		CS-	0.13	0.12
	Mean Day1	CS+	0.26	0.13
		CS-	0.17	0.12

Table S3. Paired comparison of eCBs and AA during acquisition. Two-sided paired sample t-tests revealed an increase in AEA and AA concentration during acquisition training.

**Paired comparison of eCBs and AA during acquisition**							
plasma concentration (N=45)	timepoint	*M [pmol/mL]*	*SE [pmol/mL]*	T	df	*p*	*d*
**AEA**	T1	.2945	0.017	-4.151	44	<0.001***	-0.619
[pmol/mL]	T2	.3667	0.024				
**2-AG**	T1	1.101	0.086	0.45	44	0.655	0.067
[pmol/mL]	T2	1.075	0.0801				
**AA**	T1	3190.63	259.56	-5.03	44	<0.001***	-0.749
[pmol/mL]	T2	4789.24	418.45				

*Note. The label * marks results if p < .05, the label ** marks results if p < .01, the label *** marks results if p < .001*

Table S4. Paired comparison of eCBs and AA between T3 and T4. Control analysis of plasma concentrations twenty-four hours after acquisition training when participants were waiting for 60min (similar time window as between T1 and T2). Paired comparisons of AEA, 2-AG and AA plasma (N=22) before ingestion of a placebo pill (T3) and 60min after ingestion of a placebo pill (T4) with means and standard deviation.

**Paired comparison of eCBs and AA between T3 and T4**							
plasma concentration (N=22)	timepoint	*M [pmol/mL]*	*SE [pmol/mL]*	T	*df*	*p*	*d*
**AEA**	T3	0.285	0.020	0.10	20	0.919	0.02
[pmol/mL]	T4	0.285	0.019				
**2AG**	T3	1.157	0.154	0.61	20	0.548	0.13
[pmol/mL]	T4	1.080	0.109				
**AA**	T3	3062.55	339.19	-0.96	20	0.349	-0.21
[pmol/mL]	T4	3224.07	304.35				

Note. The label * marks results if $p < .05$, the label ** marks results if $p < .01$

Table S5. Correlation analysis of eCBs and AA with acquisition effect. Pearson correlation between AEA, 2-AG and AA plasma concentrations during acquisition training (T2-T1) and fear ratings, US-expectancy ratings and SCR (CS+-CS-) during acquisition. Each p-value for each plasma concentration is corrected for three measurements of the conditioned response using the Bonferroni-Holmes method.

**Correlation analysis of eCBs and AA with acquisition effect**											
Acquisition (T2-T1)			Endocannabinoids								
			AEA			2-AG			AA		
measure	CS	N	*r*	p_{uncorr}	p_{corr}	*r*	p_{uncorr}	p_{corr}	*r*	p_{uncorr}	p_{corr}
Fear Ratings	CS+-CS-	45	0.198	0.193	0.193	0.372	0.012*	0.036*	0.222	0.142	0.184
	CS+	45	0.232	0.126		0.306	0.041*		0.315	0.035*	
	CS-	45	0.014	0.926		-0.188	0.216		0.106	0.490	
US-expectancy	CS+-CS-	41	- 0.043	0.791	>0.99	0.346	0.027*	0.054	-0.030	0.850	0.850
	CS+	41	0.017	0.916		0.250	0.115		0.019	0.905	
	CS-	41	0.093	0.565		-0.280	0.076		0.074	0.645	
SCR	CS+-CS-	42	-0.122	0.443	>0.99	-0.088	0.579	>0.99	-0.091	0.567	>0.99
	CS+	42	-0.220	0.162		-0.273	0.080		-0.207	0.189	
	CS-	42	0.020	0.898		-0.171	0.278		0.008	0.961	

*Note. The label * marks results if p < .05, the label ** marks results if p < .01, the label *** marks results if p < .001*

Table S6. Correlation of STAI with measures of acquisition. Pearson Correlation between the State Trait Anxiety Inventory (STAI) (both trait and state scores) and changes in AEA, 2-AG and AA plasma concentration during acquisition training (T2-T1), behavioral measures of conditioned responses (CS+-CS- in fear ratings, US-expectancy ratings and SCR) and AEA, 2-AG and AA plasma concentration prior to fear acquisition training. Depicted p-values are not corrected for multiple comparisons.

Correlation of STAI with measures of acquisition						
			STAI T		STAI S	
timepoints	measure	N	*r*	*p*	*r*	*p*
Acquisition (T2-T1)	AEA	45	0.288	0.055	0.147	0.337
	2-AG	45	-0.028	0.853	-0.097	0.527
	AA	45	0.334	0.025*	0.193	0.203
	Fear Ratings	45	-0.109	0.477	-6.359e -4	0.997
	US-expectancy	41	-0.352	0.024 *	-0.069	0.669
	SCR	42	0.081	0.610	-0.016	0.920
Baseline (T1)	AEA	45	0.066	0.665	0.102	0.504
	2-AG	45	0.665	0.191	0.504	0.056
	AA	45	0.068	0.657	0.073	0.634

Note. The label * marks results if $p < .05$, the label ** marks results if $p < .01$

Table S7. Correlation of US-intensity and US-valence with measures of acquisition. Pearson correlation between US-intensity and US-valence with changes in AEA, 2-AG and AA plasma concentration (T2-T1) during acquisition training, behavioral measures of conditioned responses (CS+-CS- in fear ratings, US-expectancy ratings and SCR) and AEA, 2-AG and AA plasma concentration prior to fear acquisition training. Depicted p-values are not corrected for multiple comparisons.

**Correlation of US-intensity and US-valence with measures of acquisition**						
			US-intensity		US-valence	
timepoint	measure	N	*r*	*p*	*r*	*p*
Acquisition (T2-T1)	AEA	45	0.037	0.818	-0.195	0.227
	2-AG	45	-0.063	0.695	0.087	0.595
	AA	45	-0.139	0.387	-0.006	0.972
	Fear Ratings	45	-0.193	0.204	0.216	0.159
	US-expectancy	41	-0.039	0.607	-0.169	0.297
	SCR	42	-0.171	0.278	0.119	0.457
Baseline (T1)	AEA	45	-0.017	0.917	0.050	0.760
	2-AG	45	-0.047	0.772	0.132	0.415
	AA	45	-0.048	0.763	0.0472	0.774

Note. The label * marks results if $p < .05$, the label ** marks results if $p < .01$

Table S8. fMRI main effects of acquisition. Main effects of neural responses during acquisition training, reflecting the conditioned threat response (CS+>CS-), as well as the US. Results are solely depicted for calculated regions of interest analyses, namely amygdala, dorsal ACC, insula cortex and hippocampus.

fMRI main effects of acquisition						
main effects	ROIs	T	*p* FEW corrected	*P* uncorr	z	Coordinates [MNI]
CS+ > CS-	L Amygdala	4.32	0.008 **	<0.001	3.88	x=-26 y=-8 z=-12
	R Amygdala	4.88	0.002 **	<0.001	4.28	x=26 y=-12 z=-12
	L Insula	6.42	<0.001 ***	<0.001	5.27	x=-33 y=6 z=8
	R Insula	7.7	<0.001 ***	<0.001	5.97	x=34 y=18 z=4
	dACC	7.28	<0.001 ***	<0.001	5.75	x=-4 y=28 z=26
	R Hippocampus	4.44	0.013 *	<0.001	3.97	x=22 y=-38 z=0
US	L Amygdala	4.59	0.004 **	<0.001	4.08	x=-18 y=0 z=-20
	R Amygdala	5.55	<0.001 ***	<0.001	4.74	x=22 y=0 z=-15
	L Insula	7.24	<0.001 ***	<0.001	5.73	x=-40 y=-4 z=-10
	R Insula	7.37	<0.001 ***	<0.001	5.80	x=39 y=4 z=-15
	dACC	7.54	<0.001 ***	<0.001	5.89	x=0 y=22 z=30
	L Hippocampus	5.32	0.001 ***	<0.001	4.58	x=-21 y=-26 z=-12
	R Hippocampus	4.28	0.02*	<0.001	3.85	x=20 y=-16 z=-14

Note. The label * marks results if $p < .05$, the label ** marks results if $p < .01$

Table S9. Regressor effects (AEA, 2-AG and AA – positive and negative) in fMRI regression model on association between neural responses and changes in eCBs and AA during acquisition training.

**fMRI multiple regression: CS+ > CS- with eCBs and AA during acquisition**						
measure	ROIs	T	*p* FEW corrected	*P* uncorr	z	coordinates
AEA positive	R Amygdala	3.89	0.03 *	<0.001***	3.53	x=27<br>y=-4<br>z=-16

Note. The label * marks results if *p* < .05, the label *** marks results if *p* <0.001

Table S10. Regressor effects (AEA, 2-AG and AA – positive and negative) in fMRI regression model on association between modeled linear temporal dynamics of neural responses towards the US and changes in eCBs and AA during acquisition training.

**fMRI modelled linear temporal dynamics of US with eCBs and AA during acquisition**						
measure	ROIs	T	*p* FEW corrected	*P* uncorr	z	coordinates
AEA positive	dACC	4.89	0.008 **	<0.001	4.25	x= 0<br>y= 34<br>z= 27
2-AG positive	L Amygdala	3.56	0.055	0.001	3.27	x= -15<br>y= -6<br>z= -20
	L Insula	3.89	0.061	<0.001	3.53	x= -42<br>y= 6<br>z= -3
	L Hippocampus	4.7	0.008**	<0.001	4.13	x= -15<br>y= -9<br>z= -20
2-AG negative	R Amygdala	3.5	0.074	<0.001	3.22	x= 30<br>y=-2<br>z=-27

Note. The label * marks results if $p < .05$, the label ** marks results if $p < .01$

Table S11. Main effect of task during extinction. Effects of repeated measures ANOVA analysing the acquired conditioned response from fear ratings, trial-wise US-expectancy and skin conductance responses (SCR) during extinction training.

Main effects of tasks during extinction					
**effect**	**measure**	**N**	**F**	***p***	**η^2**
CS-Type	Fear Ratings	21	(1, 20) = 15.66	<0.001***	0.27
	US-expectancy	21	(1, 20) = 11.42	0.003**	0.18
	SCR	17	(1, 16) = 4.49	0.050	0.22
Time	Fear Ratings	21	(1, 20) = 12.14	0.002**	0.10
	US-expectancy	21	(1.46, 29.175) = 22.64	<0.001***	0.19
	SCR	17	(1.28, 20.67) = 35.70	<0.001***	0.69
Time * CS	Fear Ratings	21	(1, 20) = 22.64	<0.001***	0.07
	US-expectancy	21	(1.81, 20.00) = 13.69	<0.001***	0.06
	SCR	17	(0.49, 0.38) = 20.44	<0.001***	0.56

*Note. The label * marks results if p < .05, the label ** marks results if p < .01, the label *** marks results if p < .001*

Table S12. Correlation analysis of eCBs and AA with extinction effect. Pearson correlation between AEA, 2-AG and AA plasma concentrations during extinction training (T5-T4) and fear ratings, US-expectancy ratings and SCR (CS+-CS-) during extinction. Each p-value for each plasma concentration is corrected for three measurements of the conditioned response using the Bonferroni-Holmes method.

**Correlation analysis of eCBs and AA with extinction effect**											
Extinction (T5-T4)			Endocannabinoids								
			AEA			2-AG			AA		
measure	CS	N	*r*	p_{uncorr}	p_{corr}	*r*	p_{uncorr}	p_{corr}	*r*	p_{uncorr}	p_{corr}
Fear Ratings	CS+-CS-	21	-0.303	0.182	0.546	0.198	0.389	0.389	0.269	0.239	0.478
	CS+	21	0.137	0.554		0.037	0.874		0.109	0.638	
	CS-	21	0.238	0.298		-0.267	0.242		0.133	0.565	
US-expectancy	CS+-CS-	21	-0.061	0.794	>0.99	-0.461	0.036*	0.108	-0.374	0.095	0.190
	CS+	21	0.128	0.579		-0.053	0.821		0.028	0.903	
	CS-	21	-0.025	0.915		-0.387	0.083		-0.084	0.719	
SCR	CS+-CS-	17	0.125	0.632	>0.99	-0.136	0.603	>0.99	0.237	0.360	>0.99
	CS+	17	-0.166	0.525		-0.106	0.687		0.148	0.572	
	CS-	17	-0.296	0.249		0.027	0.918		-0.085	0.747	

*Note. The label * marks results if p < .05, the label ** marks results if p < .01, the label *** marks results if p < .001*

Table S13. Main effect of task during retrieval-test. Effects of repeated measures ANOVA analysing the acquired conditioned response from fear ratings, trial-wise US-expectancy and skin conductance responses (SCR) during retrieval-test.

Main effects of tasks during retrieval-test					
**effect**	**measure**	**N**	**F**	***p***	**η^2**
CS-Type	Fear Ratings	21	(1, 20) = 14.79	0.001***	0.28
	US-expectancy	21	(1, 20) = 11.04	0.003**	0.19
	SCR	17	(1, 16) = 11.38	0.004**	0.10
Time	Fear Ratings	21	(1, 20) = 2.20	0.154	0.02
	US-expectancy	21	(1, 20) = 6.56	0.019*	0.07
	SCR	17	(1, 16) = 7.80	0.013*	0.22
Time * CS	Fear Ratings	21	(1, 20) = 6.95	0.016*	0.03
	US-expectancy	21	(1, 20 = 6.19	0.022*	0.04
	SCR	17	(1, 16) = 5.12	0.038*	0.02

*Note. The label * marks results if p < .05, the label ** marks results if p < .01, the label *** marks results if p < .001*

Table S14. Correlation analysis of retrieval effects of task and eCBs and AA effects of extinction (T5-T4). Pearson correlation between fear ratings, US-expectancy ratings and SCR (CS+-CS-) during retrieval with AEA, 2-AG and AA plasma concentrations during extinction training (T5-T4) on the second day. Each p-value for each plasma concentration is corrected for three measurements of the conditioned response using the Bonferroni-Holm method.

**Correlation analysis of retrieval effects of task and eCBs and AA extinction**												
**Retrieval** (retrieval – T5)			**Endocannabinoids Extinction(T5-T4)**									
			AEA			2-AG			AA			
measure	CS	N	*r*	p_{uncorr}	p_{corr}	*r*	p_{uncorr}	p_{corr}	*r*	p_{uncorr}	p_{corr}	
Fear Ratings	CS+-CS-	21	-0.064	0.783	0.783	0.340	0.132	0.264	0.464	0.034*	0.120	
	CS+	21	0.014	0.952		0.175	0.448		0.425	0.055		
	CS-	21	0.150	0.517		-0.238	0.299		0.092	0.691		
US-expectancy	CS+-CS-	21	-0.022	0.924	0.924	0.302	0.184	0.552	0.080	0.731	>0.99	
	CS+	21	-0.042	0.856		0.096	0.679		0.003	0.989		
	CS-	21	-0.039	0.866		-0.336	0.136		-0.129	0.576		
SCR	CS+-CS-	17	-0.027	0.919	>0.99	0.021	0.937	0.937	0.548	0.023*	0.069	
	CS+	17	-0.310	0.226		0.281	0.274		0.262	0.309		
	CS-	17	-0.430	0.085		0.395	0.117		-0.352	0.166		

*Note. The label * marks results if p < .05, the label ** marks results if p < .01, the label *** marks results if p < .001*
